# Supplementary material for: Outcome Prediction Based on Automatically Extracted Infarct Core Image Features in Patients with Acute Ischemic Stroke
Source: Diagnostics (Basel). 2022 Jul 23;12(8):1786. doi: 10.3390/diagnostics12081786 (PMC9331690; doi:10.3390/diagnostics12081786)
Supplement: Supplementary file 1 [file diagnostics-12-01786-s001.zip › diagnostics-1790288-supplementary.pdf]

Supplementary Materials

Table S1 Baseline and follow-up characteristics of the selected study and total population of the MR CLEAN NO-IV trial

Characteristic	Study population MR CLEAN NO-IV (n = 206)	Total population MR CLEAN NO-IV (n = 539)
Male sex, n (%)	111 (54)	305 (57)
Age, mean (SD)	69 (13)	70 (13)
Pre-stroke mRS >0, n (%)	46 (22)	164 (30)
EVT allocation, n (%)	97 (47)	273 (41)
Left sided stroke, n (%)	115 (56)	290 (54)
Thrombus location, n (%)		
ICA<br>M1<br>M2	48 (23)<br>123 (60)<br>34 (17)	118 (22) (n=539)<br>330 (61) (n=539)<br>85 (16) (n=539)
Other	1 (0.5)	5 (0.9) (n=539)
Follow-up infarct volume (mL), median (IQR)	17 (7.3 to 67)	11 (6 to 74)
mRS≤2, n (%)	115 (56%)	270 (50%)
